# Use of benzodiazepines and related drugs and the risk of dementia: A review of reviews

**DOI:** 10.1192/j.eurpsy.2021.1128

**Published:** 2021-08-13

**Authors:** P. Ferreira, A.R. Ferreira, L. Fernandes

**Affiliations:** 1 Fmup, Faculty of Medicine of Porto University, Porto, Portugal, Portugal, Porto, Portugal; 2 Cintesis – Center For Health Technology And Services Research, Faculty of Medicine, University of Porto, Porto, Portugal; 3 Cintesis – Center For Health Technology And Services Research; Department Of Clinical Neuroscience And Mental Health, Faculty of Medicine of Porto University; Centro Hospitalar Universitário de São João, Porto, Portugal, Portugal; 4 Cintesis – Center For Health Technology And Services Research; Department Of Clinical Neuroscience And Mental Health, Faculty of Medicine of Porto University, Porto, Portugal

**Keywords:** dementia, Cognitive decline, Benzodiazepines, Z-drugs

## Abstract

**Introduction:**

Benzodiazepines (BZDs) and related drugs (BZRDs) are widely used to reduce agitation, anxiety and sleep disturbances in the elderly, despite concerns raised about their modest efficacy for such indications and risk of severe adverse effects, including acute consequences on cognition. Recently, some studies have also raised concerns about the long-term effect of BZDs, suggesting their association with an increased risk of cognitive decline and dementia.

**Objectives:**

To review published synthesis studies on the risk of dementia development due to BZDs/BZRDs use.

**Methods:**

An electronic search was conducted in PubMed. Meta-analysis, systematic and non-systematic reviews examining the association between BZDs/BZRDs and subsequent dementia were included. No language nor publication date restrictions were applied. Search results other than synthesis studies were excluded. Studies were screened for relevance based on predefined inclusion and exclusion criteria.

**Results:**

Overall, 246 results were obtained. After initial screening, nine studies were included. From these, three were systematic reviews with meta-analysis of observational studies (cohort and/or case-control), one was a systematic review from observational studies and five were non-systematic reviews. Most studies found an association between BZDs/BZRDs and subsequent dementia, with meta-analysis studies reporting an increased risk (OR) between 1,38 and 1,78, even after controlling for protopathic bias. However, difficulties in establishing a causal relationship are reported due to the considerable clinical and methodological heterogeneity of the primary studies.

**Conclusions:**

Most studies suggest an association between the use of BZDs/BZRDs and dementia risk, highlighting that their prescription should be cautious, prevented or reduced to attenuate this risk.

